# Highly efficient thin-film 930 nm VCSEL on PDMS for biomedical applications

**DOI:** 10.1038/s41598-023-27589-1

**Published:** 2023-01-11

**Authors:** Ohdo Kwon, Sunghyun Moon, Yeojun Yun, Yong-hyun Nam, Nam-heon Kim, Donghwan Kim, Wonjin Choi, Sungjun Park, Jaejin Lee

**Affiliations:** 1 Department of Electrical and Computer Engineering, Ajou University, Suwon, 16499 South Korea; 2 RayIR Corporation, LTD, 156 Gwanggyo-ro, Yeongtong-gu, Suwon, 16506 South Korea

**Keywords:** Engineering, Optics and photonics

## Abstract

Recently, biocompatible optical sources have been surfacing for new-rising biomedical applications, allowing them to be used for multi-purpose technologies such as biological sensing, optogenetic modulation, and phototherapy. Especially, vertical-cavity surface-emitting laser (VCSEL) is in the spotlight as a prospective candidate for optical sources owing to its low-driving current performance, low-cost, and package easiness in accordance with two-dimensional (2D) arrays structure. In this study, we successfully demonstrated the actualization of biocompatible thin-film 930 nm VCSELs transferred onto a Polydimethylsiloxane (PDMS) carrier. The PDMS feature with biocompatibility as well as biostability makes the thin-film VCSELs well-suited for biomedical applications. In order to integrate the conventional VCSEL onto the PDMS carrier, we utilized a double-transfer technique that transferred the thin-film VCSELs onto foreign substrates twice, enabling it to maintain the p-on-n polarity of the conventional VCSEL. Additionally, we employed a surface modification-assisted bonding (SMB) using an oxygen plasma in conjunction with silane treatment when bonding the PDMS carrier with the substrate-removed conventional VCSELs. The threshold current and maximum output power of the fabricated 930 nm thin-film VCSELs are 1.08 mA and 7.52 mW at an injection current of 13.9 mA, respectively.

## Introduction

Optoelectronics has been extensively investigated and developed in biomedical industries for versatile applications such as optical-based biosensing, photodynamic therapy, fluorescence imaging, and laser surgery^1,2^. Particularly, biocompatible optical sources have recently attracted tremendous attention in the biomedical technology owing to their potential as next-generation medical applications for enabling it to obtain real-time monitoring information such as blood pressure, calorie consumption, and electrocardiogram (ECG).

Vertical cavity surface-emitting laser (VCSEL) has been rapidly emerging as a promising optical source compared to conventional light-emitting diodes (LEDs) and edge-emitting laser diodes (EELs) due to its low-threshold, low-divergence beam size, excellent reliability, and low-power consumption^3–6^. Furthermore, it is feasible to fabricate two-dimensional (2D) laser arrays, enabling it to pack easily into optical chips such as photonic integrated circuits (PIC). With the technological advancement for versatile VCSEL applications, numerous studies have suggested the integration of the conventional VCSELs with biocompatible polymers such as polyethylene terephthalate (PET) as well as rigid substrates such as Si and sapphire^7–10^. However, an absence of effective technology to integrate the conventional VCSELs with polymer suitable for biological tissue has limited the realization of biocompatible high-efficiency thin-film VCSELs.

Polydimethylsiloxane (PDMS), which belongs to a group of polymeric organosilicon compounds, has the potential to be used as a suitable material for bio-electronics applications owing to its biocompatibility and biostability. The PDMS is also thermally stable, flexible, and lightweight, and has low manufacturing cost in comparison with other materials used for micro-device fabrication^11,12^. It has been widely used in bioelectronics such as BioMEMS, microfluidic systems, and bio optics, enabling it to alleviate the adverse effects on human tissue such as an inflammatory response^13^. The PDMS can also protect the electronic components from mechanical and environmental impacts within a wide temperature range. This feature enables the PDMS materials to be used in the bioelectronics industry, conserving semiconductor-based micro-optical devices such as waveguides, optical fibers, and lasers^14^.

However, there have been several challenges to actualizing the biocompatible thin-film VCSELs mounted on PDMS substrates due to the unfavorable features of the PDMS in terms of device fabrication and characteristics measurement. The PDMS is considerably hydrophobic throughout the overall surface, making it difficult to combine with the surface of the hydrophilic III–V epitaxial layer during the bonding process^15–17^. Additionally, the PDMS tends to swell when coupled with several reagents, interrupting the quantitative tests for chemical analysis^18,19^. Despite several successful attempts to change the nature of the PDMS from hydrophobicity to hydrophilicity, there have been some limitations such as chemical instability, large-scale manufacturing process restriction, and difficulties in maintaining hydrophilicity for an extended period of time^11,15^.

**Figure 1:**
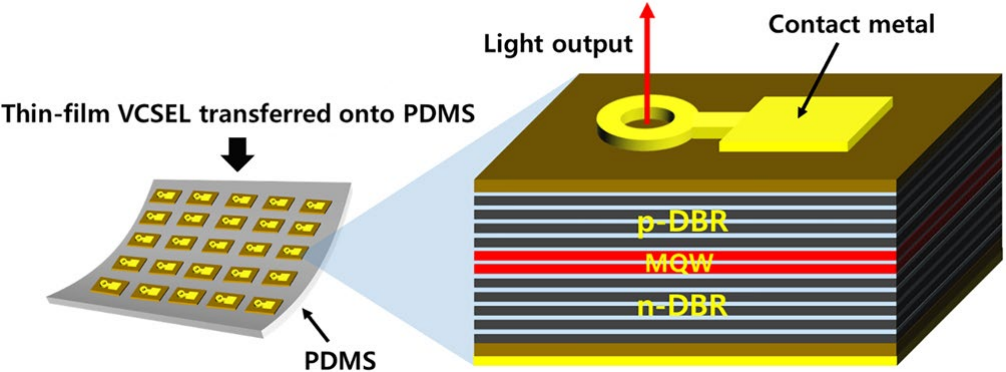
Schematic structure of the thin-film vertical-cavity surface-emitting laser (VCSEL) transferred onto a PDMS substrate. The top-emitting thin-film VCSEL was successfully fabricated onto the PDMS substrate by using a double-transfer technique in conjunction with the surface-modification bonding (SMB) process.

In this study, we successfully fabricated biocompatible thin-film 930 nm VCSELs transferred onto flexible PDMS substrates, enabling them to be used for biocompatible optical sources. In order to integrate the thin-film III–V epitaxial layer of VCSEL with the PDMS substrate, we utilized the double-transfer technique that transferred the VCSELs onto foreign carrier substrates twice for maintaining the p-on-n polarity of the thin-film VCSEL^20^. Furthermore, we employed surface modification-assisted bonding (SMB) using oxygen plasma in conjunction with organosilane treatment, which did not require any additional bonding medium when combining the PDMS carrier with substrate-removed thin-film VCSELs^21^. We also demonstrated that the transfer process of integrating the thin-film VCSEL structures onto the PDMS substrates does not seriously degrade the VCSEL performance in terms of the light–current–voltage (L–I–V) characteristics and optical spectrum^22^. Especially, we confirmed a low operating threshold current of about 1 mA for the 930 nm thin-film VCSEL at room temperature, indicating that the threshold current of the thin-film VCSELs is as low as that of the conventional VCSELs on GaAs substrate. Figure 1 shows the schematic structure of the fabricated device transferred onto the flexible PDMS substrate using the SMB and double-transfer process.

## Results and discussions

### The fabrication of 930 nm top-emitting thin-film VCSEL

**Figure 2:**
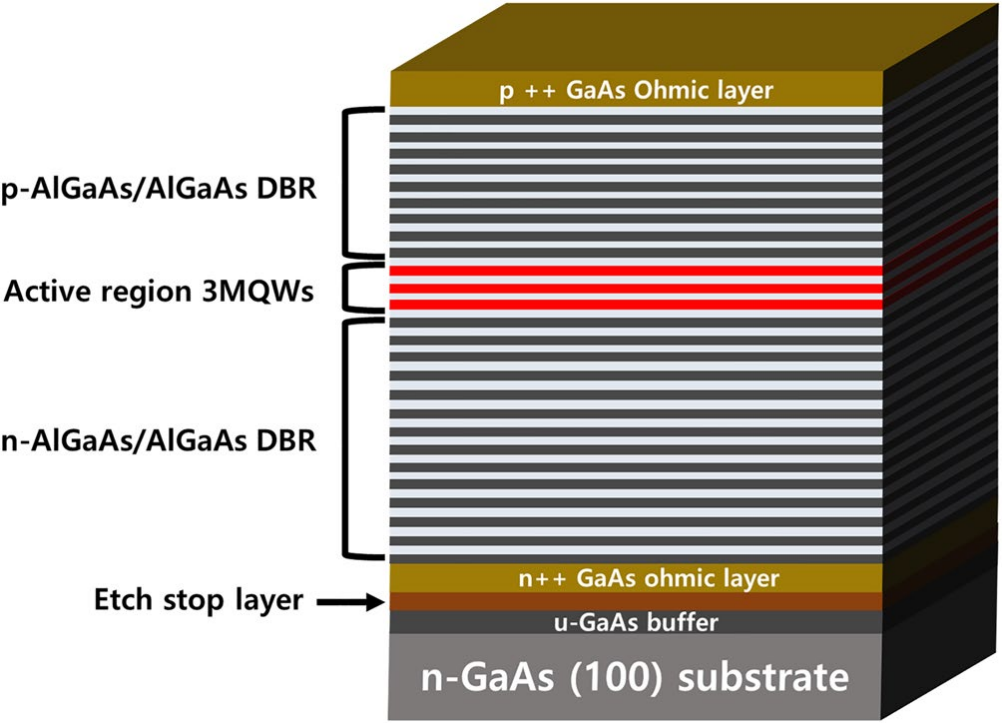
p-on-n structure of the thin-film VCSEL by MOCVD. Etch stop layer was grown on the GaAs buffer to protect the upper epitaxial layers during the removal of the GaAs substrate with the NH_4_OH-based etchant.

Figure 2 shows the epitaxially grown p-on-n structure of the thin-film VCSEL. The p-on-n structure of the thin-film VCSEL was grown on n-type GaAs substrate in upward order by metalorganic chemical vapor deposition (MOCVD). An active region of the VCSEL is composed of 3 GaAsP/InGaAs MQWs at the center sandwiched between two distributed Bragg reflectors (DBRs), consisting of n- and p-DBR alternating high and low refractive index material. Etch stop layer is grown on a GaAs buffer layer to protect VCSEL structure during GaAs substrate-removal. The heavily p- and n-doped GaAs ohmic layers serve as functional layers to alleviate the electrical loss between semiconductor and contact metal, narrowing the depletion region at the interface and enabling electrons to flow in both directions by tunneling through the barrier^23^. Additionally, the oxide aperture of the thin-film VCSEL is defined by lateral wet oxidation of AlGaAs using an electrical tube furnace. The 10-μm oxide-confined aperture can provide several merits such as the low resistance of upper DBR, the reduced recombination rate of the sidewalls near the cavity, and the less lateral current crowding outside the optical cavity^24^.

**Figure 3:**
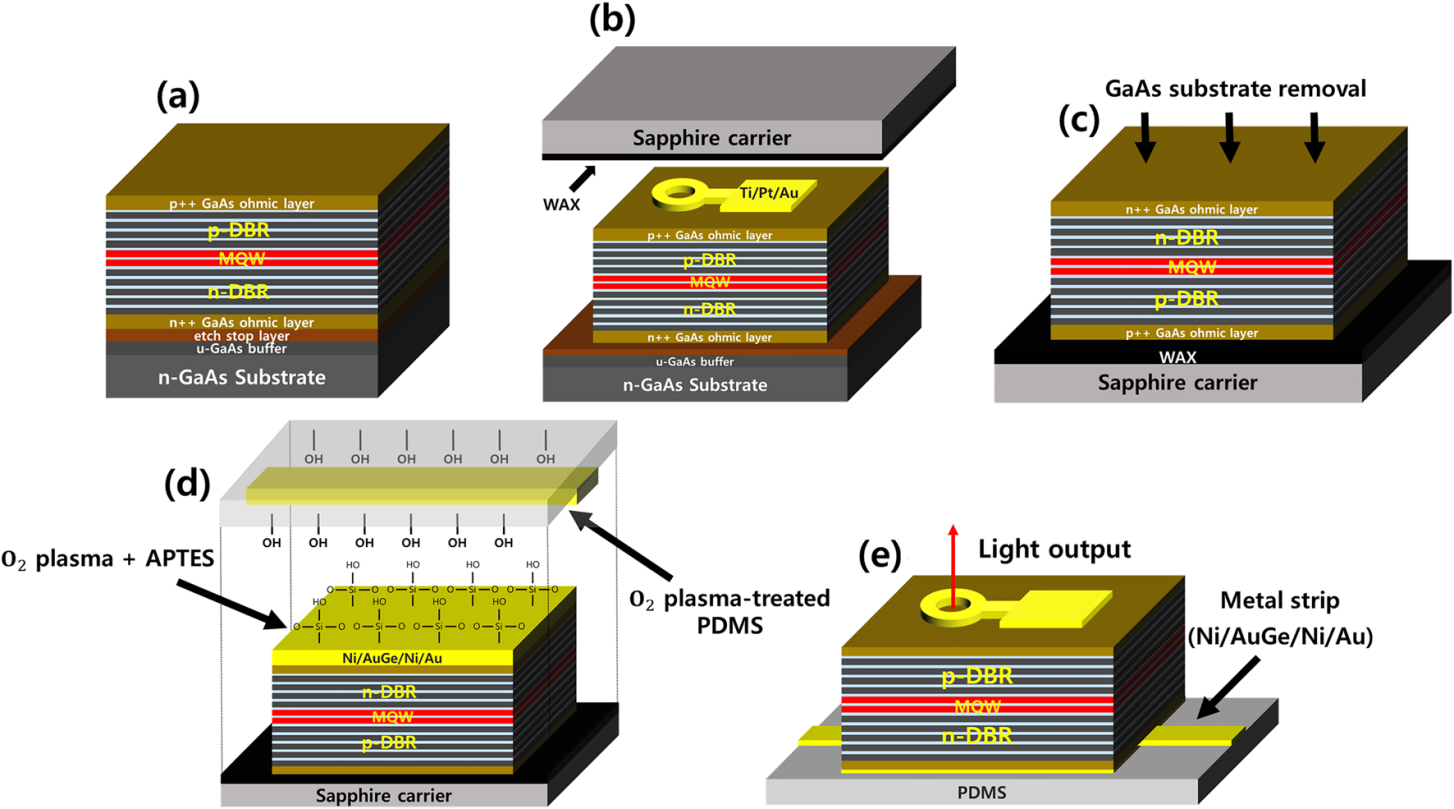
The fabrication process of the thin-film VCSEL transferred onto a PDMS substrate. (**a**) p-on-n structure of the thin-film VCSEL is grown in upward order using an MOCVD system. (**b**) The front-end process is performed including front pattern formation, top-contact (Ti/Pt/Au) metallization, and mesa etching. Subsequently, the front-processed VCSEL is bonded to the sapphire carrier using an Apiezon W wax. (**c**) The 350-μm n-GaAs substrate is removed by the NH_4_OH-based etchant. (**d**) Thin-film VCSEL with Ni/AuGe/Ni/Au is coupled to a PDMS with a metal strip via an SMB technique. (**e**) The top-emitting thin-film 930 nm VCSEL is fabricated onto a PDMS by removing the sapphire carrier.

Figure 3 shows the fabrication processes of the thin-film VCSEL transferred onto PDMS substrate using the SMB and double-transfer technique. The fabrication procedures are as follows: Fig. 3a shows the upwardly grown p-on-n structure of the thin-film VCSELs, including the heavily doped GaAs ohmic layers, a GaAs buffer layer, and an etch stop layer. Figure 3b shows a schematic of the thin-film VCSELs after performing the front-end processes, including the front pattern formation by photolithography, top-contact (Ti/Pt/Au) metallization, and mesa etching to isolate each VCSEL chip. Furthermore, Fig. 3b illustrates a process of bonding the front-processed VCSEL to the sapphire carrier for preserving the top surface on which the front-end process is completed. The top surface is attached to the sapphire carrier using an Apiezon W wax at a hot plate of about 150 °C. Figure 3c exhibits an inverted structure of the thin-film VCSELs after being mounted on the sapphire carrier. The GaAs substrate is removed with NH_4_OH-based etchant up to the etch stop layer, which prevents the etchant from penetrating the n-GaAs ohmic layer during the substrate-removal. The etch stop layer is also selectively etched by an HCl-based solution for a few seconds. Figure 3d illustrates a process of combining the inverted structure with a new PDMS carrier via an SMB technique. Before performing the bonding process, the n-contact metal (Ni/AuGe/Ni/Au) is deposited on the n-GaAs ohmic layer of the inverted structure using an e-beam evaporator. Simultaneously, a metal strip pattern (Ni/AuGe/Ni/Au) is defined on the PDMS carrier. Furthermore, the PDMS carrier is first facilitated with oxygen plasma treatment to alter the nature of PDMS from hydrophobicity to hydrophilicity. The surface of the inverted VCSEL structure is also treated with oxygen plasma followed by a 3-aminopropyltriethoxysilane (APTES) solution, enabling it to characterize as viscous adhesion and hydrophilicity^25–27^. Bonding was realized by rolling the flexible PDMS onto the inverted VCSEL structure using a roller without assistance of any bonding equipment. Figure 3e displays the fabricated top-emitting thin-film 930 nm VCSEL transferred onto the PDMS substrate.

### Transfer process description and lasing characteristics

The transfer process was first performed by bonding the sapphire carrier to the front-end processed VCSEL with an Apiezon W wax and etching the GaAs substrate with NH_4_OH-based etchant. In this procedure, the etch stop layer serves as a protective layer, preventing the etchant from permeating the epilayer of the thin-film VCSELs and enabling the complete removal of the GaAs substrate^20^. Subsequently, we performed the secondary transfer process by combining the metal layer of the sapphire-bonded inverted structure with the PDMS carrier via an SMB technique and separating it from the sapphire carrier by dissolving the Apiezon W wax using a Trichloroethylene (TCE) solution. In this bonding procedure, the surface-modification process promoted the adhesion of the PDMS with the metal layer of the thin-film VCSEL by oxygen plasma treatment.

However, the surface hydrophilicity is gradually lost because the OH– group density on the surface of the metal layer decreases after oxygen plasma treatment. Thus, the surface is coated through the APTES solution treatment after the oxygen plasma to maintain surface hydrophilicity. APTES covalently binds to the OH– group on the plasma-treated surface to form an APTES monolayer, followed by the surface covered with an amine (–NH2) group. The molecular structure of APTES, which is relatively long compared to the OH– group, can help to keep the hydrophilicity, preserving the surface chain structure.

Also, the surface coating of the thin-film VCSELs through an APTES treatment facilitated the enhancement of the adhesive strength by forming the strong siloxane (Si–O–Si) bond onto the metal layer of the thin-film VCSEL. In terms of the quantitative analysis of the SMB strength, the bonding force was evaluated between Au and PDMS through the tensile strength measurement, indicating a considerably sturdy and stable value of approximately 300 kilopascal^21^. Furthermore, the FE-SEM image of the fabricated thin-film VCSEL does not show defective structures such as cracks and dislocations which may cause significant deterioration of the VCSEL performance. We also analyzed the surface topography profile of the fabricated thin-film VCSEL and pristine PDMS material using atomic force microscopy (AFM). As shown in Supplementary Fig. S1, the fabrication process of the thin-film VCSELs led to several ridges and grooves on the top surface of the fabricated thin-film VCSELs. However, the thin-film VCSEL has a comparatively smooth topographical roughness with a root-mean-square (RMS) value of about 2.1 nm, indicating tiny roughness differences comparable to the RMS value of the PDMS substrate.

**Figure 4:**
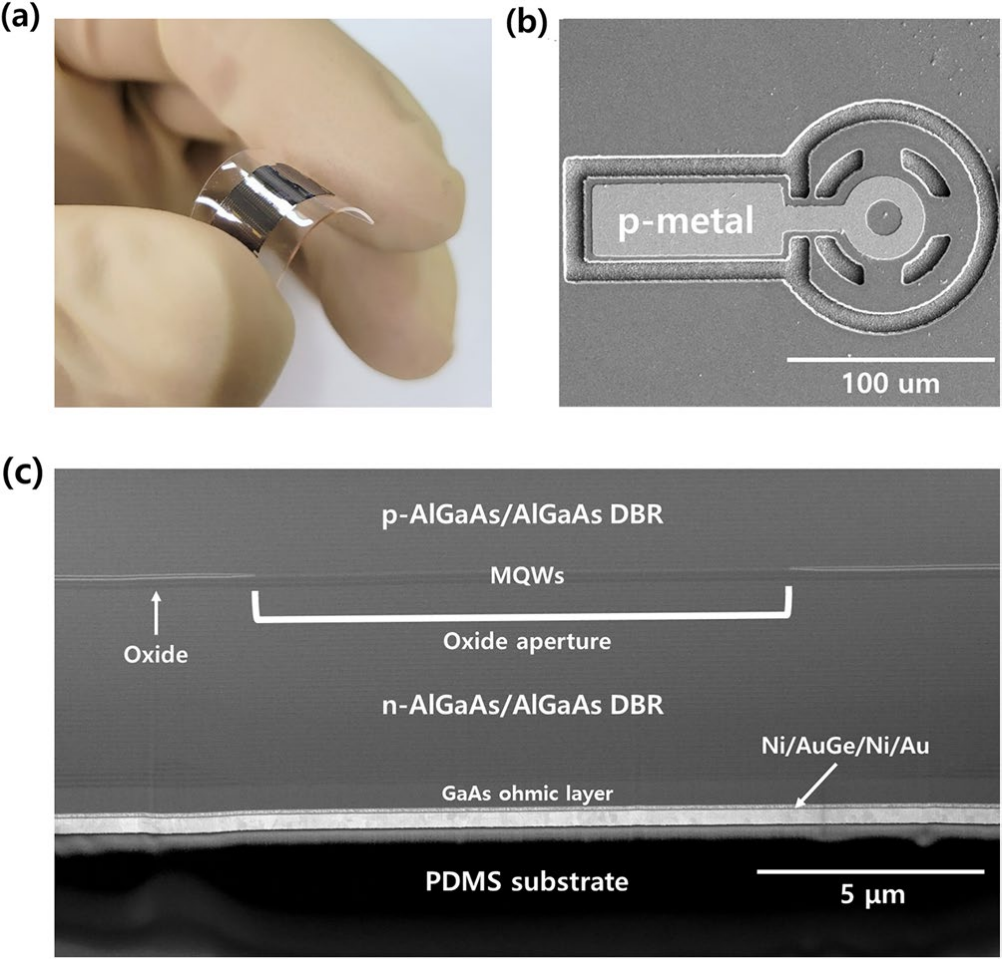
Fabrication of the top-emitting 930 nm thin-film VCSELs. (**a**) An actual image of the fabricated flexible thin-film VCSELs transferred onto PDMS, (**b**) top-view and (**c**) cross-sectional FE-SEM image of the fabricated thin-film VCSELs.

Figure 4a shows an image of the fabricated 930 nm thin-film VCSELs transferred onto PDMS, describing the thin-film VCSEL as considerably flexible. Figure 4b depicts the FE-SEM top-view of the fabricated device, exhibiting the brighter region is the p-metal used as a top-contact electrode. The 930 nm emission is from the circular aperture surrounded by p-metal on top surface of the thin-film VCSEL. Figure 4c shows the clear cross-sectional view of the thin-film VCSEL, including MQWs, p- and n-DBR, oxide aperture, n-GaAs ohmic layer, n-contact metal, and PDMS substrate. As shown in Fig. 4c, no severe crystalline defects such as voids and dislocations were observed in the cross section structure.

**Figure 5:**
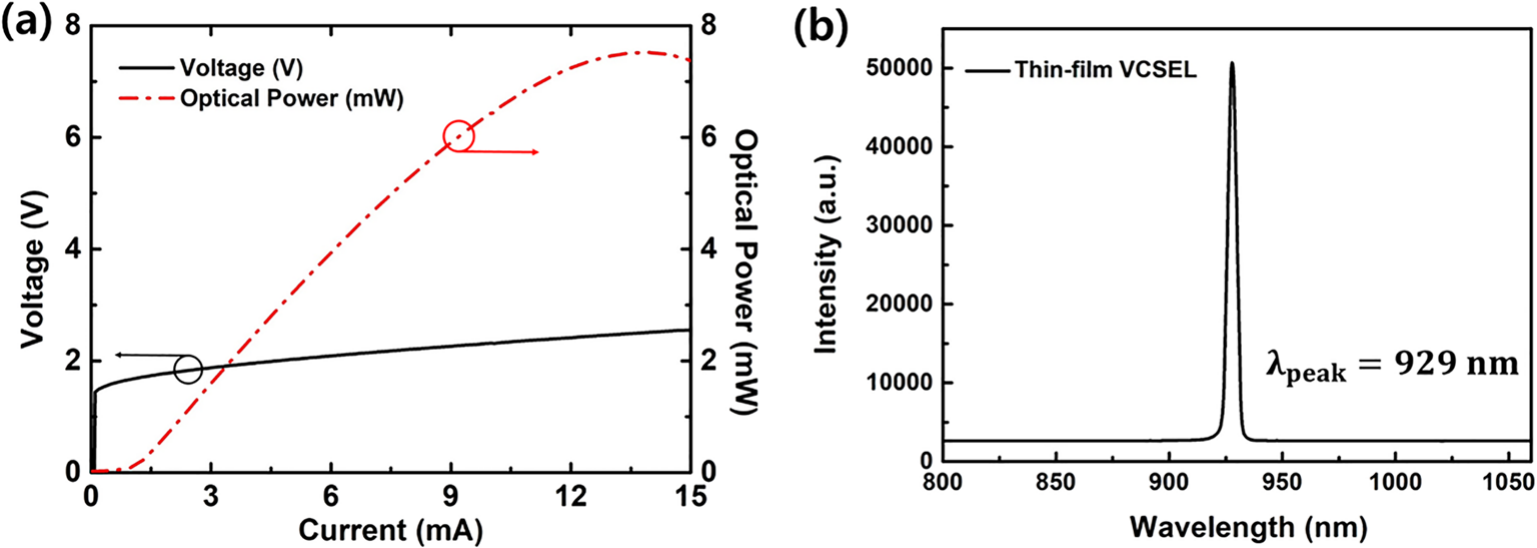
(**a**) L–I–V characteristics of the 930 nm top-emitting thin-film VCSELs onto PDMS. (**b**) Emission spectrum of the 930 nm top-emitting thin-film VCSELs. The threshold current and voltage of the fabricated thin-film VCSEL are 1.08 mA and 1.64 V, respectively. The maximum output power of the thin-film VCSEL is 7.52 mW at the injection current of 13.9 mA.

Figure 5a shows the L–I–V characteristics of the top-emitting 930 nm thin-film VCSEL transferred onto PDMS under continuous wave (CW) operation at 25 °C, indicating a threshold voltage and current of approximately 1.69 V and 1.08 mA, respectively. The maximum output power of the thin-film VCSEL is 7.52 mW at 13.9 mA of injection current. Figure 5b shows the optical spectrum from the fabricated thin-film VCSELs, indicating the peak wavelength of 929 nm.

**Figure 6:**
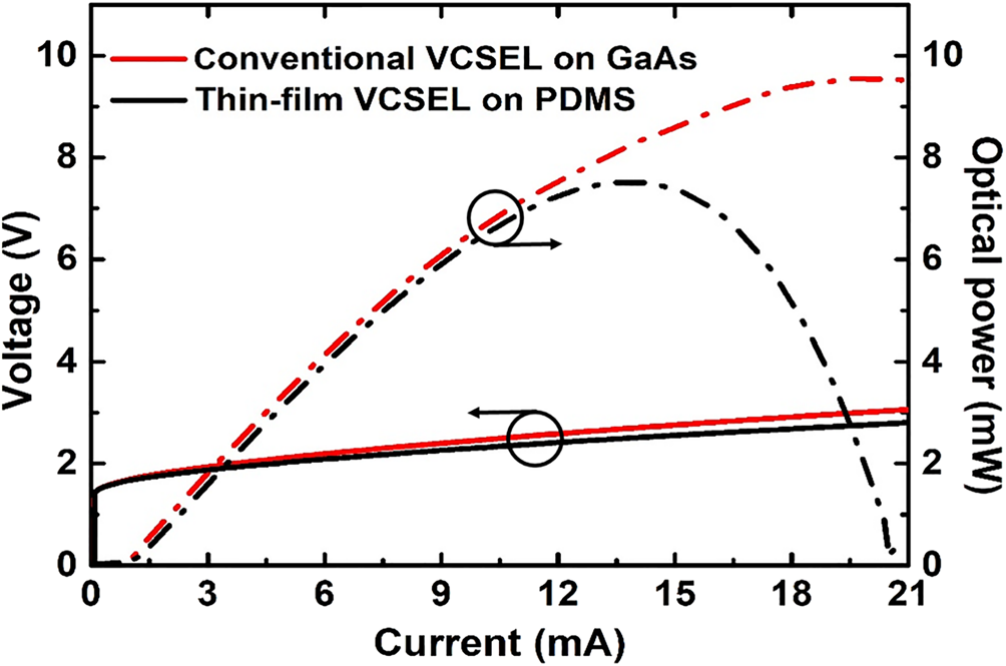
L–I–V characteristics comparison of the conventional and thin-film VCSELs. The red and black lines indicate the L–I–V characteristics of the conventional VCSEL and thin-film VCSEL mounted on PDMS, respectively. The threshold current and maximum output power of the conventional VCSEL are 1.09 mA and 9.57 mW at an injection current of 19.8 mA, respectively, while those for the thin-film VCSEL are 1.08 mA and 7.52 mW at an injection current of 13.9 mA, respectively. Furthermore, the slope efficiency of the conventional VCSEL is 0.69 W/A, and in the case of the thin-film VCSEL, the efficiency is 0.63 W/A.

As shown in Fig. 6, we compared the L–I–V characteristics between conventional VCSEL on GaAs substrate (red line) and thin-film VCSEL onto PDMS substrate (black line) under the same condition at 25 °C. The threshold current and voltage for conventional VCSEL are 1.09 mA and 1.71 V, respectively, while those for thin-film VCSEL are 1.08 mA and 1.69 V, respectively. Furthermore, the maximum output power and slope efficiency of the conventional VCSEL are 9.57 mW at an injection current of 19.8 mA and 0.69 W/A, respectively, and while these two parameters of the thin-film VCSEL are 7.52 mW at an injection current of 13.9 mA and 0.63 W/A, respectively.

The L–I–V curve of the thin-film VCSEL exhibits a lower maximum optical power compared to that of the conventional VCSEL, which is attributed to the lower thermal conductivity of the PDMS (~ 0.2 W/mK) compared to that of the GaAs substrate (~ 50 W/mK)^11,28^. Likewise, the early rollover in the thin-film VCSEL after the transfer process is ascribed to the incapability to disperse enough heat through the PDMS substrate. However, to our knowledge, the optical power of the thin-film VCSEL in this study represents a way more advanced outcome than the values of the reported research integrating the conventional VCSELs with several polymer materials^7,8^. This outcome suggests that the newly adopted double-transfer technique in conjunction with the SMB brought superior advantages in operating the thin-film VCSEL mounted onto the biocompatible PDMS polymer compared to the previous reports^7,8^.

In summary, we demonstrated the realization of biocompatible thin-film VCSELs with high flexibility transferred onto PDMS substrate. The double-transfer technique enabled the fabricated 930 nm thin-film VCSEL to maintain the p-on-n polarity. Also, the surface-modification process suggested superior bonding excellence with which any additional material is not required in order to integrate the PDMS carrier with the substrate-removed thin-film VCSELs. The maximum power of the fabricated 930 nm thin-film VCSELs on the PDMS carrier was 7.52 mW at an injection current of 13.9 mA. The measured threshold current and voltage of the thin-film VCSEL were 1.08 mA and 1.64 V, respectively. We expect this approach will open up the technological possibilities of the next-generation VCSELs for versatile biomedical applications.

## Methods

### Epitaxial growth

In this research, the VCSEL structure was epitaxially grown on the n-GaAs (100) substrate via an MOCVD system (Aix2600G3), using several precursors such as trimethylaluminum (TMAl), trimethylgallium (TMGa), trimethylindium (TMIn), arsine (AsH_3_), and phosphine (PH_3_). Carbon (CBr_4_) and silane (SiH_4_) were employed as p- and n-dopant sources, respectively. 3 InGaAs/AlGaAs MQWs were defined between the 19-pair p-DBRs and 37-pair n-DBRs. Additionally, the InGaP sacrificial layer was grown to serve as an etch stop layer between the n-GaAs ohmic contact layer and the n-GaAs buffer layer.

### L–I–V characteristics measurement

The L–I–V properties of the fabricated thin-film VCSEL were measured through a Keithley 2602B under continuous wave (CW) operation. A silicon photodiode (Hamamatsu, 2201 photodiode) was utilized to detect the 930 nm emission from the thin-film VCSELs. The emission spectrum was measured via an Ocean optics Maya 2000 PRO Spectrometer.

## Supplementary Information

Supplementary Figure S1.

## Data Availability

The datasets used and/or analysed during the current study available from the corresponding author on reasonable request.
