# Supplementary material for: Highly efficient thin-film 930 nm VCSEL on PDMS for biomedical applications
Source: Sci Rep. 2023 Jan 11;13:571. doi: 10.1038/s41598-023-27589-1 (PMC9834219; doi:10.1038/s41598-023-27589-1)
Supplement: Supplementary file 1 — Supplementary Figure S1. [file 41598_2023_27589_MOESM1_ESM.docx]

**Supplementary Information:**

**Highly efficient thin-film 930 nm VCSEL on PDMS**

**for biomedical applications**

**Ohdo Kwon^1^, Sunghyun Moon^1^, Yeojun Yun^1^, Yong-hyun Nam^1^_,_ Donghwan Kim^2^**, **Wonjin Choi^2^, Seongjun Park^1^, and Jaejin Lee^1,*^**

^1^Department of Electrical and Computer Engineering, Ajou University, Suwon, 16499, South Korea

^2^RayIR Corporation, LTD, 156 Gwanggyo-ro, Yeongtong-gu, Suwon, 16506, South Korea

^*^Corresponding author: jaejin@ajou.ac.kr

**Surface topological analysis of the fabricated top-emitting thin-film VCSELs**


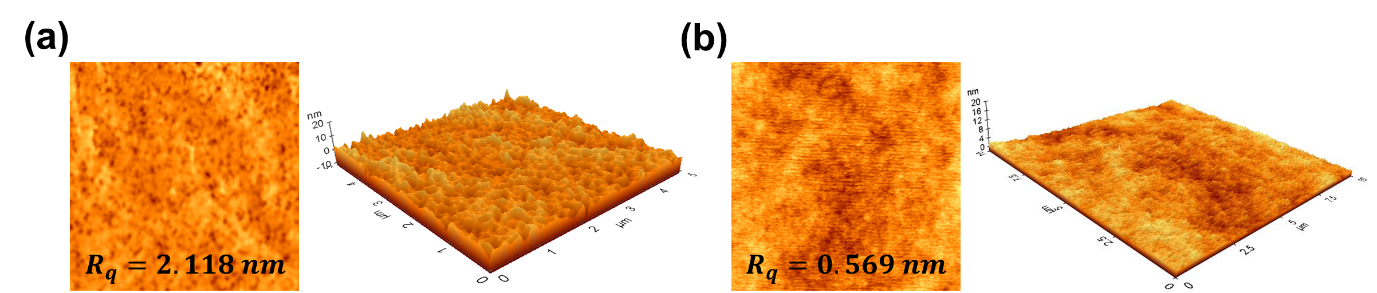


**Supplementary Fig. S1. Atomic force microscopy (AFM) 2D image (left) and 3D image (right) of (a) thin-film VCSEL transferred onto PDMS substrate and (b) PDMS material.** Supplementary Figure S1 shows the atomic force microscopy (AFM) images of the fabricated thin-film VCSELs mounted onto the PDMS and pristine PDMS. The fabricated thin-film VCSEL has a considerably smooth surface with a root-mean-square roughness (Rq) of 2.118 nm in comparison with the pristine PDMS surface with that of 0.569 nm, respectively.
